# The influence of coping, perceptions of limitations, interference, and locus of control on concussion care-seeking intentions in collegiate athletes

**Published:** 2020-04-28

**Authors:** Melissa N. Anderson, Michelle L. Weber Rawlins, Julianne D. Schmidt

**Affiliations:** ^1^Concussion Research Laboratory, Department of Kinesiology, University of Georgia, Athens, Georgia; ^2^Department of Interdisciplinary Health Sciences, A.T. Still University, Mesa, Arizona, United States

**Keywords:** mTBI, sport contact level, sport division level

## Abstract

**Background:**

Athletes who delay seeking care for a suspected concussion can experience longer recovery outcomes. Concussion care-seeking intentions may be influenced by several understudied factors; coping, perceptions of limitations, perceptions of interference, and locus of control.

**Aim:**

The aim of the study was to describe and compare coping, perceptions of limitations, perceptions of interference, and locus of control and determine whether these variables influence symptom and concussion care-seeking intentions in collegiate student-athletes.

**Methods:**

Collegiate student-athletes (n=204; female=54.9%) reported demographic information (i.e., sex, division, and contact level), symptom and concussion care-seeking intentions, coping (approach, social, and avoidance), perceptions of limitations, perceptions of interference, and locus of control ratings (internal, powerful others, and chance). Non-parametric statistics was conducted to compare all outcomes between groups (α=0.05). Multiple linear regressions were used to predict symptom and concussion care-seeking intentions based on each of the variables. Spearman rank-order correlations supplemented the regression models.

**Results:**

Females had significantly higher symptom care-seeking intentions (*P*=0.04) and greater powerful other ratings (*P*=0.04) than males. Non-contact student-athletes had significantly higher symptom care-seeking intentions (*P*<0.00) compared to collision sport athletes. Coping, perceptions of limitations, perceptions of interference, and locus of control did not significantly predict symptom or concussion care-seeking intentions. There was a weak positive association between perceptions of limitations and symptom care-seeking intentions (r_s_(198)=0.23, *P*<0.01) and concussion care-seeking intentions (r_s_(198)=0.15, *P*<0.05).

**Conclusions:**

We may not need to focus extensively on coping, perceptions of limitations, perceptions of interference, and locus of control ratings when creating concussion education since none of these variables significantly predicted care-seeking intentions.

**Relevance for Patients:**

Care-seeking intentions for concussion do not appear to be influenced largely by these variables.

## 1. Introduction

Sport-related concussions continue to present a public health burden for student-athletes at all levels of participation [1]. Although concussion rates are rising at all levels of play [2], troubling data still suggest that approximately 50% of all ­sport-related concussions go unreported and therefore undiagnosed and untreated [3-5]. Deleterious long-term effects resulting from unreported concussions may be mitigated if athletes seek timely care from a trained health-care professional. Researchers have attempted to identify reasons for the discrepancy in the estimated prevalence of concussion versus actual prevalence by examining factors such as perceived pressure from stakeholders, ability to recognize symptoms, sex disclosure, and attitudes toward care seeking [6-12]. However, most studies explain only small proportion of concussion care-seeking intentions, suggesting that this health behavior is multifactorial. To improve concussion care-seeking intentions, we must continue to determine why student-athletes choose to not seek care following concussion. This study will focus on the potential influence of three unstudied factors: Coping behaviors, perceptions of interference, perceptions of limitations, and locus of control on concussion care-seeking intentions.

Student-athletes with fewer coping resources may encounter greater difficulty with concussion care seeking. Following concussion, student-athletes often report feelings of isolation, depression, and anxiety and are often limited from or experience worsened symptoms with their usual coping resources (e.g., strenuous exercise and social interactions with peers and teammates) [13]. Inadequate coping mechanisms may influence a student-athlete to conceal his or her concussion. Similarly, student-athletes may perceive that care seeking for a suspected concussion could result in a health-care provider limiting some of their routine daily activities, such as work, school, or sports participation [6,14-16]. A falsely amplified perception of how limited they would be if they report a concussion may deter an athlete from seeking care. To the best of our knowledge, there is a current lack of literature addressing how athletes perceive limitations following concussion and how those limitations might influence their concussion care-seeking intentions. An athlete who perceives she or he may be severely limited while completing both social and physical activities following a concussion may be less likely to seek medical care.

Finally, locus of control is the fundamental appraisal of one’s self and is commonly used in personality psychology [17]. A person’s “locus” is the belief that either internal (a belief that one can control one’s own life) or external factors (i.e., how much others have power or chance) control the outcomes in their lives. Locus of control is one of the four dimensions of a person’s fundamental appraisal of themselves and is predictive of behaviors (i.e., addiction and exercise) and performance (e.g., work productivity) [17,18]. Concussed student-athletes with lower ratings of the internal locus of control subscale may experience greater difficulties with self-regulating and making the difficult decision of care seeking their concussion(s), but this relationship has not been studied.

We aimed to (1) describe and compare athlete coping, perceptions of limitations, perceptions of interference, and locus of control across demographic factors (division level, contact level, and sex) and (2) determine whether these variables influence symptom and concussion care-seeking intentions in collegiate student-athletes. We hypothesize that coping, perceptions of limitations, perceptions of interference, and locus of control will be significantly different between division level, contact level, and sex. In addition, we hypothesize that coping, perceptions of limitations, perceptions of interference, and locus of control will be positively correlated with symptom and concussion care-seeking intentions.

## 2. Methods

### 2.1. Participants

Student-athletes participating in National Collegiate Athletics Association (United States) sanctioned sports at three universities across three divisions of play ([university names blinded for peer review]: Division I, [x]: Division II, [x]: Division III, [x]:) were recruited to participate in the study during their annual concussion education session administered through an online module. During this time, athletes provided demographic information (i.e., sex, sport, and division level). All participants completed an Institutional Review Board approved consent form. Athletes who consented to be part of the research study received a series of monthly surveys through text messages that were sent on the 1^st^ day of every month from July to September 2018. The three surveys from this study were administered separately across three sequential months in the following order; perceptions of limitations and perceptions of interference, Brief Cope, and locus of control. Combined survey duration was estimated to last approximately 15 min. An additional text message reminder was sent to those who did not complete survey 2 days following initial distribution. All student-athletes who completed at least 80% of the 12 monthly surveys received a small monetary incentive.

### 2.2. Measures

#### 2.2.1. Brief Cope

Participants completed the Brief Cope survey (Appendix A) [19]. This construct has been used to assess coping strategy type for patients with health-related issues such as cancer [20] as well as orthopedic injuries [13]. The Brief Cope survey consists of 28 items, with each item stating a short description of a particular way of coping with a stressor. Examples of statements include “I’ve been turning to work or other activities to take my mind off things.” Participants were instructed to rate each item or description on a 4-point Likert scale ranging from “1=I haven’t been doing this at all” to “4=I have been doing this a lot.” For this study, the collapsed scoring for the Brief Cope was used: Approach (10 items), avoidance (8 items), and social (4 items) [21] (Table 1). Each of the three subscores is computed by adding scores resulting in a range between 4 and 40.

**Table 1:** Brief cope subscore definitions.

Avoidance	Denial, venting, behavioral disengagement, self-blame
Social	Emotional support, instrumental support
Approach	Planning, positive reframing, humor, religion, and active coping

#### 2.2.2. Perceptions of limitations

We developed a survey to assess how limited an athlete thought that they would be after sustaining a concussion (Appendix B). Participants reported how limited they believed they would be following a concussion with an 11-item measure (e.g., attending class and working at a job) on a 5-point Likert scale with responses ranging from “1=not at all limited” to “5=extremely limited.” Scores were summed across all items with a higher score implicating the belief of being extremely limited following a concussion.

#### 2.2.3. Perceptions of interference

Participants reported how much they believed partaking in a list of activities would interfere with concussion recovery with a 15-item measure (e.g., socially drinking, using recreational drugs) (Appendix B). Participants responded on a 5-point Likert scale with responses ranging from “1=none at all” to “5=a great deal.” Responses were summed together to get a total perception of interference score. A higher total score suggested a greater degree of perceived interference that their life would have on concussion recovery.

The perceptions of limitations and perceptions of interference survey tools were developed by the lead author and reviewed for content and face validity by coauthors who have expertise in concussion. Reliability of the survey was conducted among 30 university students who completed the survey 2 times approximately 2 weeks apart, revealing high internal consistency and reliability for perceptions of limitations (11 items, a=0.95, ICC_2,1_=0.92) and perceptions of interference (15 items, a=0.97, ICC_2,1_=0.94).

#### 2.2.4. Locus of control

Locus of control was measured using Levenson’s Multidimensional Locus of Control scale (Blau, 1984). This 6-point Likert scale (1=“strongly disagree,” 6=“strongly agree”) survey includes 24 items (Appendix C) separated into the following subscores: Internal control, powerful others, and chance (Table 2). The internal control (I) scale measures the extent to which an individual believes that they have control over the outcomes in their lives. An example of a statement specific to internality is “When I make plans, I am almost certain to make them work.” The powerful others (P) scale deals with how much control an individual believes that other people in power have control over the outcomes in their lives, for example, “In order to make plans work, I make sure they fit in with the desires of the people who have power over me.” Finally, the chance (C) scale addresses the extent to which an individual believes the outcomes in their lives is up to fate and they have no control over what happens, for example, “It is not wise for me to plan too far ahead because many things turn out to be a matter of good or bad luck.”

**Table 2:** Locus of control subscore definitions.

Internality	The belief that you largely have control over the outcomes in your own life
Powerful others	The belief that your fate is controlled by other people
Chance	The belief that your fate is controlled by chance

#### 2.2.5. Concussion care-seeking intentions

Participants completed a survey on their symptom care-seeking intentions and concussion care-seeking intentions (Appendices D and E). All items were scored using a 7-point Likert scale with responses ranging from 1=“strongly disagree” to “7=“strongly agree.” Eight questions addressed symptom care-seeking intentions (e.g., “I would stop playing and report my symptoms if I sustained an impact that caused me to see stars”) and the remaining three questions assessed concussion care-seeking intentions (e.g., “I intend to report”). Responses were summed for concussion care-seeking intentions (three items) and symptom care-seeking intentions (eight items) with a higher score indicating better care-seeking intentions on each measure of intentions. These measures were derived by Kroshus *et al*. (2016) and Register-Mihalik (2013) and have high internal consistency (Cronbach’s a=0.89) [5,7,22].

#### 2.2.6. Analyses

To address our first aim, frequencies and descriptive statistics were used to describe sex, division, and contact level. Student-athletes were classified into three categories for their sport (i.e., collision, limited contact, and non-contact) [23]; however, collision and limited-contact groups were later collapsed for analyses where sample size was not sufficient. Kruskal–Wallis tests were used to determine if Brief Cope scores, perceptions of interference, perceptions of limitations, or locus of control ratings differed between divisions (I, II, and III), and level of contact (contact and non-contact). Mann–Whitney *U*-tests were used to compare whether there were differences between males and females for Brief Cope scores (three dependent variables of avoidance, approach, and social), perceptions of interference and limitations, and locus of control ratings (three dependent variables of internal control, powerful others, and chance).

To address our second aim, multiple linear regressions were calculated to predict symptom and concussion care-seeking intentions based on Brief Cope scores, perceptions of interference and limitations, and locus of control ratings. A series of Spearman rank-order correlations were also conducted to determine if coping, perceptions of limitations, perceptions of interference, and locus of control were positively correlated with symptom and concussion care-seeking intentions. Data were analyzed using IBM SPSS Statistics (Version 24.0.00, IBM Corp., Armonk, NY) with an a priori a=0.05.

## 3. Results

### 3.1. Demographics

A total of 204 student-athletes (92 males [45.1%] and 112 females [54.9%]) completed at least one of the three surveys. Student-athlete descriptive and demographic data are presented in Table 3.

**Table 3:** Survey results across sex, division level, and contact level.

	Sex			Contact				Division level			
		
	Median (IQR)			Median (IQR)				Median (IQR)			
		
	Male	Female	*P*	Collision	Limited contact	Non-contact	*P*	DI	DII	DIII	*P*
Symptom care-seeking intentions	5.50 (2.21)	6.00 (1.37)	0.04^*^	5.62 (2.13)	5.87 (1.63)	5.86 (1.94)	0.00^*^	5.56 (2.19)	5.25 (2.12)	6.00 (1.13)	0.64
Concussion care- seeking intentions	6.00 (2.00)	6.00 (1.25)	0.51	6.50 (1.50)	6.00 (1.58)	6.00 (1.67)	0.22	6.00 (1.91)	6.33 (2.00)	6.67 (1.33)	0.82
Brief Cope											
Avoidance	12.00 (5.00)	13.00 (6.00)	0.76	15.50^**^ (6.25)		12.00 (4.25)	0.07	13.00 (6.00)	13.00 (6.00)	13.00 (6.00)	0.73
Social	11.00 (4.00)	11.00 (3.00)	0.91	11.50^**^ (3.00)		10.00 (4.00)	0.05^*^	10.00 (3.50)	9.00 (4.50)	11.00 (5.00)	0.13
Approach	27.00 (8.00)	26.00 (7.50)	0.56	28.5^**^ (6.25)		26.00 (7.00)	0.02^*^	27.00 (5.00)	23.00 (8.00)	27.00 (8.00)	0.21
Perceptions of limitations	52.00 (12.00)	54.00 (9.00)	0.43	48.00 (10.50)	53.00 (8.50)	54.00 (10.00)	0.04^*^	51.50 (11.00)	55.00 (11.00)	54.00 (9.00)	0.73
Perceptions of interference	28.00 (15.00)	25.00 (19.00)	0.43	29.50 (18.50)	27.50 (14.25)	26.00 (17.25)	0.33	29.00 (20.75)	28.00 (23.50)	25.00 (13.00)	0.13
Locus of control											
Internality	34.00 (9.75)	34.00 (7.00)	0.85	33.00^**^ (9.00)		34.00 (6.00)	0.06	34.00 (7.50)	33.00 (6.25)	35.00 (6.00)	0.20
Powerful others	21.00 (16.00)	17.25 (12.00)	0.04^*^	19.00^**^ (16.50)		20.50 (13.00)	0.84	23.00 (13.75)	19.50 (13.25)	20.00 (13.00)	0.27
Chance	16.00 (11.50)	16.00 (10.00)	0.23	16.00^**^ (9.75)		16.00 (12.00)	0.18	16.50 (13.00)	19.50 (11.50)	16.00 (7.00)	0.46

* Indicates significance at 0.05 level

** indicates collapsing of collision and limited collision sports due to small sample size

### 3.2. Results from perceptions of limitations and perceptions of interference survey

**Figure 1:**
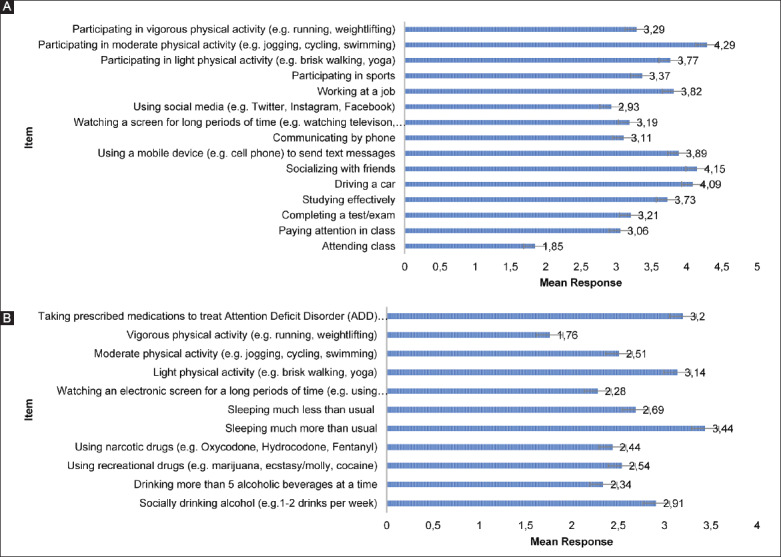
Mean responses for the (A) perceptions of limitations and (B) perceptions of interference surveys.

Student-athletes most commonly perceived that a concussion would extremely limit their ability to complete a test or examination (extremely limited=119/212, 56.1%) and participate in sports (extremely limited=127/211, 59.9%). Student-athletes most commonly perceived that a concussion would interfere a great deal with their ability to watch an electronic screen for a long period of time (e.g., using mobile devices and watching television) (would interfere a great deal=75/212, 35.4%) and perform vigorous physical activity (would interfere a great deal=119/212, 56.1%). Nearly, all student-athletes (n=198/203, 97.5%) responded that all 14 activities on the perceptions of limitations survey would be hindered at least somewhat by a concussion and 97.2% of student-athletes responded that every item on the interference survey would interfere with their concussion recovery to some degree (n=204/212). Individual item mean responses for both of the surveys are shown in Figure 1.

### 3.3. Care-seeking intentions across sex, division level, and contact level

Female student-athletes had significantly higher symptom care-seeking intentions (Table 3); however, we did not observe this difference in concussion care-seeking intentions (*P*=0.51). Symptom care-seeking intentions (*P*=0.64) and concussion care-seeking intentions (*P*=0.82) did not significantly differ between division levels. Student-athletes who participated in non-contact sports and limited-contact sports had significantly higher symptom care-seeking intentions (*P*<0.001) than student-athletes in collision sports; however, there were no significant differences between concussion care-seeking intentions and contact level (*P*=0.22). All comparisons between groups and care-seeking intentions are presented in Table 3.

### 3.4. Group comparisons for sex, contact level, and division level

Contact athletes reported higher ratings of social (*P*=0.05) and approach (*P*=0.02) coping than non-contact athletes. Non-contact athletes reported that a concussion would much more greatly limit their daily activities than contact athletes (*P*=0.04). Finally, female student-athletes reporting significantly greater powerful other ratings (*P*=0.04) than males. There were no significant differences between division level and any of the survey responses (*P*>0.05). All group comparisons are presented in Table 3.

### 3.5. Relationship with care-seeking intentions

**Figure 2:**
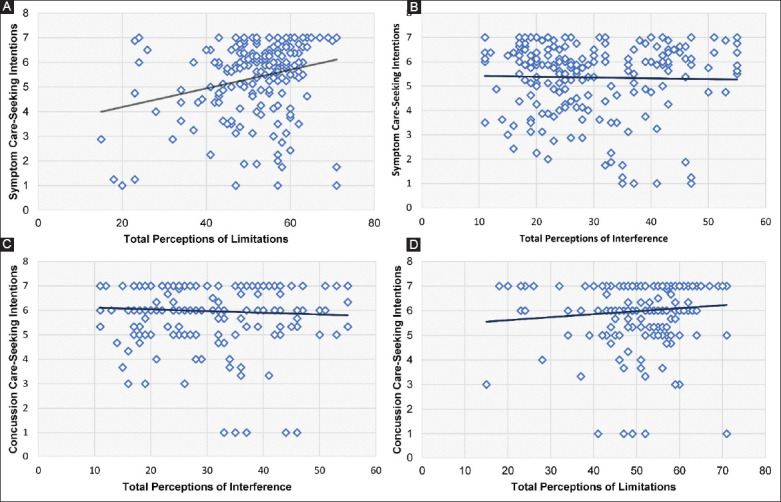
Total perceptions of limitations and symptom care-seeking intentions (A), total perceptions of interference and symptom care-seeking intentions (B), total perceptions of interference and concussion care-seeking intentions (C), total perceptions of limitations and concussion care-seeking intentions (D).

Only student-athletes who completed all three surveys were included in regression analysis (n=68/202; 33.67%). Brief Cope subscores, perceptions of limitations, perceptions of interference, and locus of control subscores, did not significantly predict concussion care-seeking intentions but accounted for 33% of the variance (F(8,68)=1.03, *P*=0.43, R^2^=0.33). In addition, none of the independent variables significantly predicted symptom care-seeking intentions but accounted for 23% of the variance (F(8,68)=0.47, *P*=0.87, R^2^=0.23). We observed a weak positive association between perceptions of limitations and symptom care-seeking intentions (r_s_(198)=0.23, *P*<0.01) and concussion care-seeking intentions (r_s_(198)=0.15, *P*<0.05) (Figure 2). However, no other correlations yielded significant associations and are presented in Table 4.

**Table 4:** Spearman’s rank-order correlations.

			Care-seeking intentions	

			Concussion	Symptom
Brief Cope	Avoidance	*n*	100	100
		ρ	−0.17	−0.60
		*P*	0.87	0.56
	Social	*n*	100	100
		ρ	−0.05	0.31
		*P*	0.61	0.75
	Approach	*n*	99	99
		ρ	−0.05	−0.21
		*P*	0.59	0.83
Perceptions of limitations		*n*	199	199
		ρ	0.15	0.23
		*P*	0.03^*^	0.00^*^
Perceptions of interference		*n*	199	199
		ρ	−0.05	−0.04
		*P*	0.51	0.56
Locus of control	Internality	*n*	123	123
		ρ	0.05	0.08
		*P*	0.60	0.41
	Powerful others	*n*	123	123
		ρ	−0.13	−0.12
		*P*	0.14	0.19
	Chance	*n*	123	123
		ρ	−0.07	−0.06
		*P*	0.45	0.48

* Correlation is significant at the 0.05 level (two tailed)

## 4. Discussion

Overall, the key finding of this study is that care-seeking intentions for students-athletes were not significantly predicted by any of the subscore on the Brief Cope, locus of control, or the perceptions of limitations and perceptions of interference survey. However, our research did yield several interesting findings, specifically differences between males and females and contact level, outlined in the following sections. This study supports existing literature that found that many factors influence student-athletes intentions to report a suspected concussion to sports medicine professionals [6-8,11,12,14-16,24].

### 4.1. Care-seeking intentions and survey response differences between males and females

Females had higher ratings on the locus of control powerful others subscore than males. This implies that female student-athletes felt that they had less control over the outcomes in their own lives than their male student-athlete counterparts. Females tend to have larger social support networks and, therefore, may be more inclined to believe that external factors such as powerful others have more control over the outcomes in their lives [25]. It is important to note that higher ratings on the external locus of control scale do not necessarily imply worse health outcomes and have actually been documented to be advantageous [25]. Females are more likely to seek medical care which may result in earlier diagnosis, treatment, and better prognoses [22,26,27]. Specifically, female athletes have been documented to be more likely to report concussion symptoms to an authoritative figure [10-12,22] and the current study yielded similar results with our finding that female student-athletes had significantly higher intentions to seek care for their concussion symptoms than males.

### 4.2. Differences between contact levels

Differences in personality characteristics between contact and non-contact student-athletes have been well-documented [28-30] and may explain differences we observed between contact levels and responses for care-seeking intentions [22]. Individuals tend to self-select participating in activities that meet their training needs with aggressive people actively seeking contact sport and less aggressive people choosing to participant in non-contact sport [29]. Contact student-athletes reported being more likely to use social and approach coping behaviors than non-contact athletes. Contact athletes often self-report as being more combative on and off the field [30], are more extraverted [28], and perceive anger to be facilitative to their performance [29]. This tendency toward aggression may explain why contact athletes are more likely to utilize approach coping behaviors rather than avoidance like their non-contact sport counterparts. These characteristics are then often reinforced within the sport. Non-contact and limited-contact student-athletes had significantly higher symptom care-seeking intentions than contact athletes (Table 3). In addition, limited- and non-contact student-athletes believed that they would be significantly more limited following a concussion than contact athletes. This difference between contact levels could explain why student-athletes in these groups also had greater symptom care-seeking intentions than athletes in collision sports.

Despite not reaching significance, we found an interesting trend between contact level and internality ratings for locus of control (*P*=0.06). Contact student-athletes reported on average lower mean internality ratings than non-contact athletes (Table 3). This finding indicates that contact athletes may not believe that they have as much control over the outcomes in their lives compared to non-contact athletes. Many contact sports rely on working with teammates to achieve an objective (e.g., scoring a goal) and, regardless of the strength of an individual athlete, the final outcome of the event depends on the actions of the group, not the individual [30]. Conversely, non-contact sports are often based on the performance of the individual (e.g., cross-country, tennis, swim, and dive) [29,31] with these athletes reporting higher internal locus of control ratings [17,32].

#### 4.2.1. Perceptions of limitations and perceptions of interference

Student-athletes reported that, in general, a concussion would at least somewhat limit their ability to complete all 14 activities listed on the perceptions of limitations survey. Specifically, athletes reported that they would be most limited in the classroom and completing school-specific activities with nearly 60% of athletes reporting that a concussion would extremely limit their ability to take a test. Based on these concerns related to academic performance following concussion, it is critical that we educate athletes and stakeholders on the importance of return to learn after concussion as well as make sure that academic accommodations and adjustments are provided when needed [33]. Athlete perceptions of limitations scores were weakly correlated with both symptom and concussion care-seeking intentions. This finding indicates that the degree to which a student-athlete believes a concussion would interfere with their everyday life may have a small influence on their intentions to seek care. However, it should be noted that these correlation values were weak and perceptions of limitations were not a predictor within our larger regression model. Continuing to educate all athletes on how concussion symptoms can affect their day-to-day life may, in turn, positively influence the number of athletes who seek care following a suspected injury. Individuals who are more knowledgeable about the severity of an injury or illness are significantly more likely to seek medical care [27]. Sports medicine professionals should continue to educate all athletes on the serious nature of concussions and possible ramifications of failing to report an injury (e.g., increased number of days before returning to play [34,35]).

### 4.3. Limitations

This study is not without limitations. Our care-seeking intentions survey was administered pre-season at a single time point and the three follow-up surveys were distributed over the course of 1 year. It is possible that student-athletes care-seeking intentions evolved and changed overtime. While we accounted for concussions that occurred over the course of the data collection period, we did not account for a previous history of concussion which could have inflated an athlete’s knowledge of the injury. Specifically, previous exposure to concussion may alter an athlete perception of how limited they would be following a concussion and may have greatly influenced their survey responses [36]. In addition, our sample of Division I, II, and III student-athletes was limited to a specific part of the United States and, therefore, our findings may not be generalizable to other regions.

## 5. Conclusions

The results of our study reveal that many student-athletes believe that a concussion would limit their ability to do well at a variety of sport-related and non-sport-related activities. Our findings are clinical and translation due to our integration of psychological constructs to help explain concussion-reporting intentions in a sample of college-aged student-athletes. There are several significant differences between contact level and care-seeking intentions which need to be further investigated to determine how much this influences actual concussion-reporting behaviors. Our findings indicate that while these things may not influence concussion reporting and we might not need to focus on them extensively in concussion education. By doing so, a multifaceted approach to increase care-seeking intentions can be implemented which may lead to better recovery outcomes.

### Disclosure Statement and Conflicts of Interest

Melissa Anderson and Dr. Michelle Weber Rawlins have received stipend and travel funds from The National Collegiate Athletic Association – Department of Defense Research Grand Challenge: Changing Attitudes about Concussions in Young and Emerging Adults Grant.

Dr. Julianne Schmidt has received research funding from The National Collegiate Athletic Association – Department of Defense Research Grand Challenge: Changing Attitudes about Concussions in Young and Emerging Adults Grant.

## Appendix A: Brief Cope

These items deal with ways you have been coping with the stress in your life since you found out you were going to have to have this operation. There are many ways to try to deal with problems. These items ask what you have been doing to cope with this one. Obviously, different people deal with things in different ways, but I am interested in how you have tried to deal with it. Each item says something about a particular way of coping. I want to know to what extent you have been doing what the item says. How much or how frequently. Do not answer on the basis of whether it seems to be working or not – just whether or not you are doing it. Use these response choices. Try to rate each item separately in your mind from the others. Make your answers as true FOR YOU as you can.

1=I have not been doing this at all

2=I have been doing this a little bit

3=I have been doing this a medium amount

4=I have been doing this a lot

1. I have been turning to work or other activities to take my mind off things
2. I have been concentrating my efforts on doing something about the situation I am in
3. I have been saying to myself “this isn’t real”
4. I have been using alcohol or other drugs to make myself feel better
5. I have been getting emotional support from others
6. I have been giving up trying to deal with it
7. I have been taking action to try to make the situation better
8. I have been refusing to believe that it has happened
9. I have been saying things to let my unpleasant feelings escape
10. I have been getting help and advice from other people
11. I have been using alcohol or other drugs to help me get through it
12. I have been trying to see it in a different light, to make it seem more positive
13. I have been criticizing myself
14. I have been trying to come up with a strategy about what to do
15. I have been getting comfort and understanding from someone
16. I have been giving up the attempt to cope
17. I have been looking for something good in what is happening
18. I have been making jokes about it
19. I have been doing something to think about it less, such as going to movies, watching TV, reading, daydreaming, sleeping, or shopping
20. I have been accepting the reality of the fact that it has happened
21. I have been expressing my negative feelings
22. I have been trying to find comfort in my religion or spiritual beliefs
23. I have been trying to get advice or help from other people about what to do
24. I have been learning to live with it
25. I have been thinking hard about what steps to take
26. I have been blaming myself for things that happened
27. I have been praying or meditating
28. I have been making fun of the situation.

## Appendix B1

Perceptions of limitations: Please rate how much you think partaking in the following actions would interfere with recovery following a concussion:

1=A great deal

2=A lot

3=A moderate amount

4=A little

5=None at all

1. Socially drinking alcohol (e.g., 1-2 drinks/week)
2. Drinking more than 5 alcoholic beverages at a time
3. Using recreational drugs (e.g., marijuana, ecstasy/molly, and cocaine)
4. Using narcotic drugs (e.g., oxycodone, hydrocodone, and fentanyl)
5. Sleeping much more than usual
6. Sleeping much less than usual
7. Watching an electronic screen for a long period of time (e.g., using mobile devices and watching television)
8. Light physical activity (e.g., brisk walking and yoga)
9. Moderate physical activity (e.g., jogging, cycling, and swimming)
10. Vigorous physical activity (e.g., running and weightlifting)
11. Taking prescribed medicines to treat attention-deficit disorder (ADD) or attention-deficit hyperactivity disorder (ADHD).

## Appendix B2

Perceptions of interference: Please rate how limited you think someone recovering from a concussion would be at completing the following actions:

1=A great deal

2=A lot

3=A moderate amount

4=A little

5=None at all

1. Attending class
2. Paying attention in class
3. Completing a test/exam
4. Studying effectively
5. Driving a car
6. Socializing with friends
7. Using a mobile device (e.g., cell phone) to send text messages
8. Communicating by phone
9. Watching a screen for long periods of time (e.g., watching television, using tablet, or iPad)
10. Using social media (e.g., Twitter, Instagram, and Facebook)
11. Working at a job
12. Participating in sports
13. Participating in light physical activity (e.g., brisk walking and yoga)
14. Participating in moderate physical activity (e.g., jogging, cycling, and swimming).

## Appendix C

Levenson’s multidimensional locus of control scale

For each of the following statements, indicate the extent to which you agree or disagree by circling the appropriate number.

1=Strongly disagree

2=Disagree somewhat

3=Slightly disagree

4=Slightly agree

5=Agree somewhat

6=Strongly agree

1. Whether or not I get to be a leader depends mostly on my ability
2. To a great extent my life is controlled by accidental happenings
3. I feel like what happens in my life is mostly determined by powerful people
4. Whether or not I get into a car accident depends mostly on how good a driver I am
5. When I make plans, I am almost certain to make them work
6. Often there is no chance of protecting my personal interests from bad luck
7. When I get what I want, it is usually because I am lucky
8. Although I might have a good ability, I will not be given leadership responsibility without appealing to those in positions of power
9. How many friends I have depends on how nice a person I am
10. I have often found that what is going to happen will happen
11. My life is chiefly controlled by powerful others
12. Whether or not I get into a car accident is mostly a matter of luck
13. People like myself have very little chance of protecting our personal interests when they conflict with those of strong pressure groups
14. It is not always wise for me to plan too far ahead because many things turn out to be a matter of good or bad fortune
15. Getting what I want requires pleasing those people above me
16. Whether or not I get to be a leader depends on whether I am lucky enough to be in the right place at the right time
17. If important people were to decide they did not like me, I probably would not make many friends
18. I can pretty much determine what will happen in my life
19. I am usually able to protect my personal interests
20. Whether or not I get into a car accident depends mostly on the other driver
21. When I get what I want, it is usually because I worked hard for it
22. In order to have my plans work, I make sure that they fit in with the desires of people who have power over me
23. My life is determined by my own actions
24. It is chiefly a matter of fate whether or not I have a few friends or many friends.

## Appendix D

Symptom care-seeking intentions

Please rate how strongly you agree with the following statement: “I would stop playing and report my symptoms if I sustained an impact that caused me to.”

1=Strongly disagree

2=Disagree

3=Somewhat disagree

4=Neither agree nor disagree

5=Somewhat agree

6=Agree

7=Strongly agree

1. See stars
2. Vomit or feel nauseous
3. Have a hard time remembering things
4. Have problems concentrating on the task at hand
5. Feel sensitive to light or noise
6. Have a headache
7. Experience dizziness or balance problems
8. Feel sleepy or in a fog.

## Appendix E

Concussion Care-Seeking Intentions

Directions: Rate on how strongly you agree with the following statement: “When I experience possible concussion symptoms…”

1=Strongly disagree

2=Disagree

3=Somewhat disagree

4=Neither agree nor disagree

5=Somewhat agree

6=Agree

7=Strongly agree

1. I intend to report
2. I plan to report
3. I will make an effort to report.

## References

[1] Langlois JA, Rutland-Brown W, Wald MM (2006). The Epidemiology and Impact of Traumatic Brain Injury:A Brief Overview. J Head Trauma Rehabil.

[2] Zuckerman SL, Kerr ZY, Yengo-Kahn A, Wasserman E, Covassin T, Solomon GS (2015). Epidemiology of Sports-Related Concussion in NCAA Athletes from 2009-2010 to 2013-2014:Incidence, Recurrence, and Mechanisms. Am J Sports Med.

[3] Llewellyn T, Burdette GT, Joyner AB, Buckley TA (2014). Concussion Reporting Rates at the Conclusion of an Intercollegiate Athletic Career. Clin J Sport Med.

[4] McCrea M, Barr WB, Guskiewicz K, Randolph C, Marshall SW, Cantu R (2005). Standard Regression-based Methods for Measuring Recovery after Sport-related Concussion. J Int Neuropsychol Soc.

[5] Register-Mihalik JK, Guskiewicz KM, McLeod TC, Linnan LA, Mueller FO, Marshall SW (2013). Knowledge, Attitude, and Concussion-Reporting Behaviors Among High School Athletes:A Preliminary Study. J Athl Train.

[6] Kerr ZY, Register-Mihalik JK, Kroshus E, Baugh CM, Marshall SW (2016). Motivations Associated With Nondisclosure of Self-Reported Concussions in Former Collegiate Athletes Motivations Associated With Nondisclosure of Self-Reported Concussions in Former Collegiate Athletes. Am J Sports Med.

[7] Kroshus E, Baugh CM, Daneshvar DH, Viswanath K (2014). Understanding Concussion Reporting Using a Model Based on the Theory of Planned Behavior. J Adolesc Health.

[8] Kroshus E, Garnett B, Hawrilenko M, Baugh CM, Calzo JP (2015). Concussion under-Reporting and Pressure from Coaches, Teammates, Fans, and Parents. Soc Sci Med.

[9] Milroy JJ, Hebard S, Kroshus E, Wyrick DL (2018). Sport-Related Concussion Reporting and Coach-Athlete Attachment among Collegiate Student-Athletes. J Clin Sport Psychol.

[10] Miyashita TL, Diakogeorgiou E, VanderVegt C (2016). Gender Differences in Concussion Reporting Among High School Athletes. Sports Health Multidiscip Approach.

[11] Sullivan L, Pursell L, Molcho M (2018). Evaluation of a Theory-based Concussion Education Program for Secondary School Student-athletes in Ireland. Health Educ Res.

[12] Wallace J, Covassin T, Beidler E (2017). Sex Differences in High School Athletes'Knowledge of Sport-Related Concussion Symptoms and Reporting Behaviors. J Athl Train.

[13] Kontos AP, Elbin RJ, Appaneal RN, Covassin T, Collins MW (2013). A Comparison of Coping Responses among High School and College Athletes with Concussion, Orthopedic Injuries, and Healthy Controls. Res Sports Med.

[14] Chrisman SP, Quitiquit C, Rivara FP (2013). Qualitative Study of Barriers to Concussive Symptom Reporting in High School Athletics. J Adolesc Health.

[15] Delaney JS, Lamfookon C, Bloom GA, Al-Kashmiri A, Correa JA (2015). Why University Athletes Choose Not to Reveal Their Concussion Symptoms During a Practice or Game. Clin J Sport Med.

[16] Delaney Caron JG, Correa JA, Bloom GA (2018). Why Professional Football Players Chose Not to Reveal Their Concussion Symptoms During a Practice or Game. Clin J Sport Med.

[17] Ajzen I (2002). Perceived Behavioral Control, Self-Efficacy, Locus of Control, and the Theory of Planned Behavior. J Appl Soc Psychol.

[18] Blau GJ (1984). Brief Note Comparing the Rotter and Levenson Measures of Locus of Control. Percept Mot Skills.

[19] Carver CS (1997). You want to Measure Coping but Your Protocol's Too Long:Consider the Brief COPE. Int J Behav Med.

[20] Yusoff N (2010). Reliability and Validity of the Brief COPE Scale (English Version) Among Women with Breast Cancer Undergoing Treatment of Adjuvant Chemotherapy:A Malaysian Study. Med J Malaysia.

[21] Snell DL, Siegert RJ, Hay-Smith EJC, Surgenor LJ (2011). Factor Structure of the Brief COPE in People With Mild Traumatic Brain Injury. J Head Trauma Rehabil.

[22] Weber ML, Suggs DW, Bierema L, Miller LS, Reifsteck F, Schmidt JD (2019). Collegiate Student-athlete Sex, Years of Sport Eligibility Completed, and Sport Contact Level Influence on Concussion Reporting Intentions and Behaviours. Brain Inj.

[23] Rice SG (2008). Medical Conditions Affecting Sports Participation. Pediatrics.

[24] Sanderson J, Weathers M, Snedaker K, Gramlich K (2017). I was able to Still do my Job on the Field and Keep Playing:An Investigation of Female and Male Athletes'Experiences with (not) Reporting Concussions. Commun Sport.

[25] Sherman AC, Higgs GE, Williams RL (1997). Gender Differences in the Locus of Control Construct. Psychol Health.

[26] Mansfield A, Addis M, Mahalik J (2003). Why won't he go to the Doctor?:The Psychology of Men's Help Seeking. Int J Mens Health.

[27] Taber JM, Leyva B, Persoskie A (2015). Why do People Avoid Medical Care?A Qualitative Study Using National Data. J Gen Intern Med.

[28] Elman WF, McKelvie SJ (2003). Narcissism in Football Players:Stereotype or Reality?Athl Insight.

[29] Lemieux P, McKelvie SJ, Stout D (2002). Self-reported Hostile Aggression in Contact Athletes, No Contact Athletes and Non-athletes. Athl Insight.

[30] Reuter JM (2005). The Relationships among Three Components of Perceived Risk of Injury, Previous Injuries and Gender in Non-Contact/Limited Contact Sport Athletes. Athl Insight.

[31] Nicholls AR, Polman RC, Levy AR, Backhouse SH (2009). Mental Toughness in Sport:Achievement Level, Gender, Age, Experience, and Sport Type Differences. Personal Individ Differ.

[32] Lynn RW, Phelan JG, Kiker VL (1969). Beliefs in Internal-External Control of Reinforcement and Participation in Group and Individual Sports. Percept Mot Skills.

[33] Master CL, Gioia GA, Leddy JJ, Grady MF (2012). Importance of “Return-to-Learn”in Pediatric and Adolescent Concussion. Pediatr Ann.

[34] Asken BM, McCrea MA, Clugston JR, Snyder AR, Houck ZM, Bauer RM (2016). “Playing Through It”:Delayed Reporting and Removal From Athletic Activity After Concussion Predicts Prolonged Recovery. J Athl Train.

[35] Elbin RJ, Sufrinko A, Schatz P, French J, Henry L, Burkhart S (2016). Removal From Play After Concussion and Recovery Time. Pediatrics.

[36] Schmidt JD, Lynall RC, Lempke LB, Weber ML, Devos H (2018). Post-concussion Driving Behaviors and Opinions:A Survey of Collegiate Student-athletes. J Neurotrauma.

